# Successful Sirolimus Treatment for Recurrent Pericardial Effusion in a Large Cervicomediastinal Provisionally Unclassified Vascular Anomaly: A Case Report

**DOI:** 10.1055/a-2057-7177

**Published:** 2023-05-17

**Authors:** Julio César Moreno-Alfonso, María San Basilio Berenguer, María del Carmen Sarmiento Caldas, Jesús González Cayón, Santiago de la Puente, Paloma Triana, Juan Carlos López-Gutiérrez

**Affiliations:** 1 Department of Pediatric Surgery, Hospital Universitario de Navarra, Pamplona, Spain; 2 Doctoral School of Navarre, Universidad Pública de Navarra, Pamplona, Spain; 3 Department of Pediatric Surgery, Hospital Infantil La Paz, Madrid, Spain; 4 Department of Pediatric Surgery, Hospital Universitario Reina Sofía, Córdoba, Spain; 5 Department of Pediatric Surgery, Hospital Universitario Niño Jesús, Madrid, Spain; 6 Department of Pediatric Plastic Surgery, Hospital Infantil La Paz, Madrid, Spain

**Keywords:** vascular malformations, provisionally unclassified vascular anomaly, sirolimus, pericardial effusion, case report

## Abstract

Provisionally unclassified vascular anomalies (PUVA) are a group of diseases with unique characteristics that make them unclassifiable within vascular tumors or malformations. We describe a PUVA as the cause of recurrent pericardial effusion and its response to sirolimus. A 6-year-old girl was referred with a cervicothoracic vascular anomaly, a violaceous, and irregular lesion in the neck and upper chest, diagnosed as “hemangioma”. She had pericardial effusion at the neonatal age that required pericardiocentesis, propranolol, and corticosteroids. She remained stable for 5 years, when she presented with a severe pericardial effusion. A magnetic resonance visualized a diffuse vascular image in the cervical and thoracic region with mediastinal extension. The pathological study showed a vascular proliferation in the dermis and hypodermis with positive staining for Wilms' Tumor 1 Protein (WT1) and negative for Glut-1. Genetic testing found a variant in *GNA14* , for which the diagnosis of PUVA was established. When a pericardial drain was placed without response, treatment with sirolimus was started with resolution of the effusion. Sixteen months later, the malformation is stable and there has been no recurrence of pericardial effusion. In a significant group of patients, definitive diagnosis is not possible despite pathological and genetic analysis. Mammalian target of rapamycin inhibitors may become a therapeutic option if symptoms are severe enough, with a low rate of reported side effects.

## Introduction

Provisionally unclassified vascular anomalies (PUVAs) are a group of conditions that cannot be classified as malformations or tumors within the last International Society for the Study of Vascular Anomalies (ISSVA) classification. 1 PUVA is an heterogeneous group of anomalies including intramuscular hemangioma, fibro adipose vascular anomaly, and angiokeratoma among others. 2 3 There are also included some entities for which it is not possible to give a definitive diagnosis and therefore pose serious therapeutic difficulties. We present a case of a girl with recurrent pericardial effusion due to a cervicothoracic vascular anomaly with an excellent response to sirolimus.

## Case Report

A 6-year-old girl was referred to our Vascular Anomalies Center for a complex cervicothoracic vascular anomaly initially diagnosed as hemangioma. She was born at 38.3 weeks to a healthy mother, with a birth weight of 3,250 g and a 5-minute Apgar score of 10 with normal physical examination. In the first 12 hours of life, she presented tachypnea and arterial hypotension; therefore, chest X-ray and echocardiogram were performed showing a pericardial effusion that required pericardiocentesis, obtaining 40 mL of serous fluid. A thoracic magnetic resonance imaging (MRI) demonstrated a bilateral submandibular vascular anomaly with thoracic and mediastinal extension suggestive of a “deep hemangioma” as the cause of the effusion. Propranolol was started with no response, requiring further pericardiocentesis at 48 hours of life, after which corticosteroid was added and the effusion resolved. The patient evolved favorably and was discharged at 25 days of life with 2 mg/kg/day of propranolol and oral prednisolone (1.5 mg/kg/day) for 12 months.

**Fig. 1:**
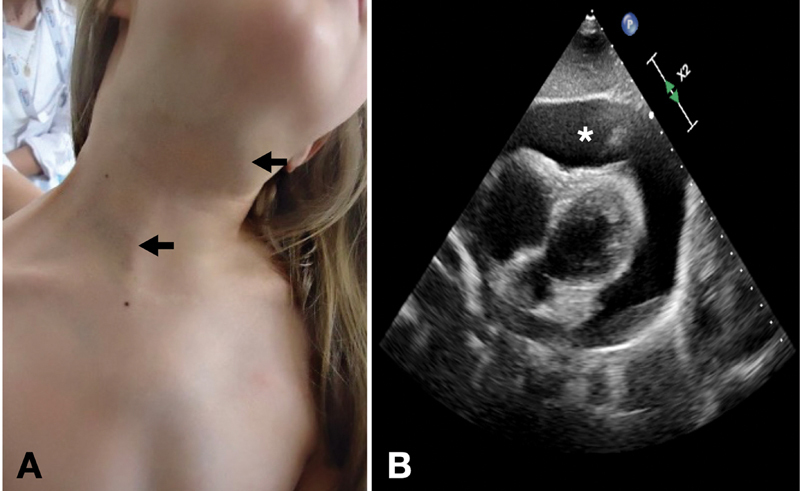
( **A** ) Vascular anomaly with subcutaneous cervical component and thoracic extension with slight violaceous skin coloration and minimal superficial component (arrows). ( **B** ) Circumferential pericardial effusion of 36 mm with mild diastolic restriction, collapse of right cavities, and echographic signs of cardiac tamponade (*).

The patient remained stable for 5 years, until the time of referral to our hospital for a pericardial effusion that did not improve despite propranolol and corticosteroids. For the last 4 months she had been suffering from recurrent tonsillitis; however, she presented in good general condition with normal vital signs, weight 37 kg and height 122 cm (body surface area 1.14 m ^2^ ). On examination, there was a subcutaneous, violaceous, and irregular lesion on the neck and upper thorax with slight superficial component ( Fig. 1A ), associated with intermittent pain and distant heart sounds; thus, an echocardiogram was performed that confirmed severe pericardial effusion impairing cardiac dynamics ( Fig. 1B ).

**Fig. 2:**
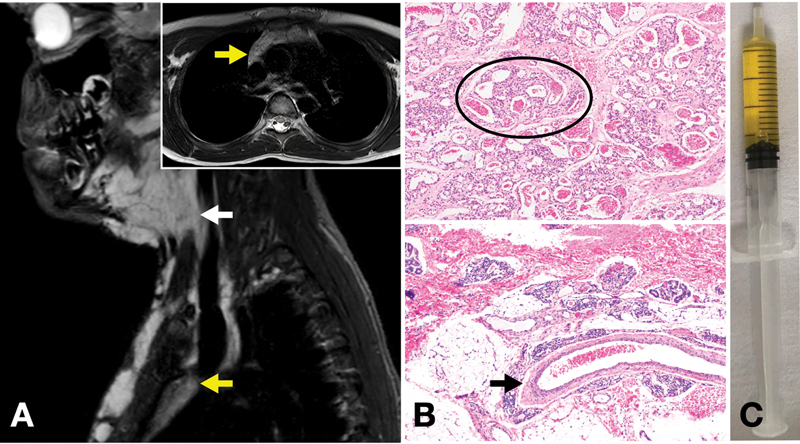
( **A** ) Diffuse vascular proliferation in the cervical region (white arrow) with thoracic and mediastinal extension (yellow arrow). ( **B** ) Hematoxylin and eosin stain x3 magnification showing on one side lobules of hypercellularity and predominance of capillaries (oval), and on the other side proliferation of dilated and large vessels in lower dermis and hypodermis with scarce cellularity (arrow). Histologic findings were compatible with characteristics of vascular tumor and, at the same time, of vascular malformation. ( **C** ) Appearance of pericardial effusion fluid.

Subsequently, an MRI was performed that visualized a diffuse vascular lesion in the subcutaneous tissues of the cervical and upper thoracic region, with extension to the anterior mediastinum in the upper two-thirds of the retrosternal area and surrounding the outlet of the great vessels ( Fig. 2A ). Intranodal lymphography was normal. The pathological analysis of an open biopsy of affected skin in the cervical area showed a vascular proliferation in dermis and hypodermis without spindle cells, positive staining for WT1, and negative for Glut-1. Genetic analysis found a variant in *GNA14* . Sample also presented different degrees of cellularity suggesting a tumor etiology, but on the other hand, an important vascular component pointing to a vascular malformation ( Fig. 2B ), so a diagnosis by exclusion of PUVA was established.

We started treatment with prednisolone (2 mg/kg/day) without improvement in the effusion. Then, a pericardial drain was placed evacuating 280 mL of lymphoid fluid with a pH 7.4, protein 1 g/dL, lactate dehydrogenase 80 IU/L, cholesterol 26 mg/dL, triglycerides 31 mg/dL, and leukocytes 200/mm ^3^ without malignant cells, compatible with transudate ( Fig. 2C ). Debit on the 6th day of placement was 20 mL/24 hours and echocardiographically there was no effusion, hence the pericardial catheter was removed. Five days later, a similar effusion occurred again; thus, a pericardiocentesis was performed and in the absence of chylous fluid and malformations susceptible to sclerosis or removal, treatment with sirolimus at a dose of 0.8 mg/m ^2^ /12 hours was indicated (2 mg/day) and the effusion improved progressively until its complete reabsorption. After 16 months of treatment, the vascular anomaly has remained clinically stable and there has been no recurrence of the effusion. The patient has not presented any side effects and is currently on sirolimus; the dose has been adjusted according to weight gain to 2.5 mg/day, maintaining sirolimus plasma levels between 15 and 18.4 ng/mL.

## Discussion

The ISSVA current classification from 2018 divides vascular anomalies into tumors and malformations. However, there is a group of pathologies that due to their particularities cannot be included in any of these two categories: PUVA. 2 This is a “junk drawer” in which vascular anomalies with unique morphological and histological characteristics are incorporated, as in the case presented.

In our case, the patient was referred with the diagnosis of hemangioma due to the imaging characteristics and given the absence of appreciable malformation at birth; therefore, propranolol and corticosteroids were administered with good response, which reinforced this diagnostic suspicion. However, our initial impression upon seeing a soft, bluish subcutaneous mass in the cervical region was a venous malformation. Due to the history of neonatal pericardial effusion and since we did not have the previous radiological studies, we performed the whole set of diagnostic images, which suggested a probable cervico-thoraco-mediastinal vascular malformation that conditioned a significant pericardial effusion. Although we have not been able to prove it, since the lymphography was normal, we believe that the effusion could be explained by some lymphatic component of the malformation, since the patient had previously had multiple pharyngeal infections, which could have originated the lymphatic transudate with pericardial effusion by contiguity. In fact, the fluid draining from the pericardium had the appearance of lymph. Precisely, we suggest that the patient remained asymptomatic during the previous years because she did not have intercurrent processes that activated or inflamed any of the components of the malformation.

After imaging studies, a pathological study was performed identifying alterations compatible with vascular malformation, although there were also some tumor characteristics, resulting inconclusive. In cases such as this, our recommendation is to perform a sufficient open biopsy of the affected tissue under sedation whenever possible. If for aesthetic reasons or comorbidities this is not possible, punch biopsies of affected tissue (ideally 4 mm) are an acceptable alternative. However, in urgent clinical situations, genetic or histological study is not essential for decision making, as it can take from weeks to months.

In the last years, the genotype of vascular anomalies has gained importance and various mutations have been identified, mainly found in the *RAS/MEK/ERK* and *PIK3CA/Akt/mTOR* signaling pathway, the former giving rise to high-flow malformations and tumors and the latter associated with low-flow vascular malformations. 4 5 Genetic testing has become, therefore, a pillar in the study of vascular anomalies, opening the door to targeted therapies and serving as a prognostic variable. 6 7 Our patient had a mutation in *GNA14* , which has been reported in tufted angioma, kaposiform hemangioendothelioma, and pyogenic granuloma, all vascular tumors in the *RAS/MEK/ERK* pathway. 8 9 Despite imaging, histopathology, and genetic testing, it was not possible to characterize a specific disease and the diagnosis of PUVA was established.

Although the magnitude of the effusion, the patient was paucisymptomatic and we decided to start with corticosteroids, but without response, therefore requiring the placement of a pericardial drain with transient improvement and early recurrence after its removal. Given the refractoriness of the condition, we initiated sirolimus, with excellent response. Sirolimus, a mammalian target of rapamycin (mTOR) inhibitor, has demonstrated its effectiveness and safety in the treatment of many vascular anomalies in the pediatric population with a low rate of severe side effects. 10 11 12 In this case, the therapy has been empirical, because despite histological and genetic studies, a definitive diagnosis could not be performed. In fact, the only mutation demonstrated was in the *RAS/MEK/ERK* pathway and yet the response to sirolimus was favorable, suggesting either that there are feedback pathways in the signaling cascade that we do not know about or that the rare vascular anomaly in our patient had multiple components, some of them responders to sirolimus. In complex vascular anomalies, such as this one, and in the absence of response to other therapies (propranolol, corticosteroids) we suggest trying sirolimus, even if there is not yet a documented genetic mutation or histological diagnosis. Due to the particularity of these cases, there is no established follow-up protocol, but we believe that after starting sirolimus patients should be assessed monthly or quarterly during the first year, with baseline sirolimus levels to guide treatment, and repeated only in case of symptoms requiring dose adjustment. Since there is a great deal of interindividual pharmacokinetic variability and scarce correlation between blood levels and therapeutic response, which means that similar concentrations of the same drug can produce different effect in each patient. 13 Similarly, imaging follow-up is determined by the type of malformation and the patient's symptoms.

There are reports of vascular malformations, mainly lymphatic, associated with recurrent pericardial effusion, but none of the pathologies included in the PUVA or treated with sirolimus. 14 15 16 However, the characteristics of the vascular anomaly described above make this a unique malformation, and we have not found similar reports to date. Probably in the coming years, as our knowledge of pathogenic and signaling pathways advances, we will be able to give a more precise name or classification to many of the entities we now call PUVA, since the genes that cause some of these disorders are known, but we do not know most of their mechanisms.

## Conclusion

In a significant group of patients with vascular anomalies, definitive diagnosis is not possible despite radiological, histological, and genetic analysis. In these cases, there is an indication for mTOR inhibitors if symptoms are severe enough considering the low rate of reported side effects with its use.

## Notes

New Insights and the Importance for the Pediatric Surgeon In patients with vascular anomalies in whom it is not possible to reach a diagnosis despite multiple tests, a safe and effective option is to try mammalian target of rapamycin inhibitors if symptoms are severe enough, considering the low rate of reported side effects with their use.
